# Nanoparticle surface stabilizing agents influence antibacterial action

**DOI:** 10.3389/fmicb.2023.1119550

**Published:** 2023-02-09

**Authors:** Thelma Ameh, Kusy Zarzosa, Jake Dickinson, W. Evan Braswell, Christie M. Sayes

**Affiliations:** ^1^Department of Environmental Science, Baylor University, Waco, TX, United States; ^2^United States Department of Agriculture, Animal and Plant Health Inspection Services, Plant Protection and Quarantine, Science and Technology, Insect Management and Molecular Diagnostics Laboratory, Edinburg, TX, United States

**Keywords:** engineered nanoparticles, stabilizing agent, minimum inhibitory concentration, alternative antimicrobial resistance agent, minimum bactericidal concentration

## Abstract

The antibacterial properties of nanoparticles are of particular interest because of their potential to serve as an alternative therapy to combat antimicrobial resistance. Metal nanoparticles such as silver and copper nanoparticles have been investigated for their antibacterial properties. Silver and copper nanoparticles were synthesized with the surface stabilizing agents cetyltrimethylammonium bromide (CTAB, to confer a positive surface charge) and polyvinyl pyrrolidone (PVP, to confer a neutral surface charge). Minimum inhibitory concentration (MIC), minimum bactericidal concentration (MBC), and viable plate count assays were used to determine effective doses of silver and copper nanoparticles treatment against *Escherichia coli*, *Staphylococcus aureus* and *Sphingobacterium multivorum*. Results show that CTAB stabilized silver and copper nanoparticles were more effective antibacterial agents than PVP stabilized metal nanoparticles, with MIC values in a range of 0.003 μM to 0.25 μM for CTAB stabilized metal nanoparticles and 0.25 μM to 2 μM for PVP stabilized metal nanoparticles. The recorded MIC and MBC values of the surface stabilized metal nanoparticles show that they can serve as effective antibacterial agents at low doses.

## Introduction

Nanoparticles are used as antibacterial agents (Morones et al., 2005; Ruparelia et al., 2008; Varshney et al., 2012). The composition of antibacterial nanoparticles varies in physical and chemical properties. Some employ organic based liposomes and capsules filled with conventional antibiotics or novel RNAs, termed nano-carriers; while others exploit the cation leaching from metal colloid surfaces as the main antibacterial agent (Zhang et al., 2010; Hallaj-Nezhadi and Hassan, 2015; Mi et al., 2018). In the latter class of nanoparticles, the metal colloid can be engineered to include different chemical compositions (such as silver or copper), surface functionalities (such as aqueous suspension stabilizing agents or surface charges), or primary particle size (<15 nm in diameter to enable passive diffusion across the bacterial cell wall and other intracellular membranes or > 50 nm to enable extended duration of cation leaching in either biological or environmental matrices). Each of these properties are compiled into a nanoparticle profile and if the antibacterial activity of each property can be measured, then antibacterial nanoparticles can be designed in a safe and effective manner.

Silver and copper nanoparticles are commercially available and are currently used as antibacterial (and in some cases antimicrobial) applications. For instance, silver particles are incorporated into bandages to prevent bacterial infections in wounded skin (Wilkinson et al., 2011; Liu et al., 2018). Copper nanoparticles are used in antifouling paints for the prevention of biofilm formation in the hull of ships (Anyaogu et al., 2008; Padmavathi et al., 2019). In some cases, the nanoparticle formed is a zero-valent metal colloid which can be described as clusters of metal ions reduced to zero valency and self-assembled into spheres coated with a blanket of positively, negatively, or neutrally charged functional groups (Li et al., 2008; An and Somorjai, 2012). Metal-based “core-shell” nanoparticles are precisely engineered for size, shape, and surface charge – three of the most useful and tailorable properties that induce cytotoxicity (Berg et al., 2009; El Badawy et al., 2011; Mélinon et al., 2014; Liu et al., 2021).

In other cases, metal nanoparticles can be produced in a crystallization process involving both a metal cation and inorganic anion (Ahmed et al., 2012). One example is copper (I) iodide (CuI). CuI are suspected to have enhanced antimicrobial activity because both copper and iodine ions are present and have the potential to leach into the surface matrix (Renné et al., 2017). Copper ions, in particular the monovalent form (Cu^+1^) is cytotoxic to bacteria, viruses, fungi, and molds due to oxidation process [i.e., from its monovalent to its divalent (Cu^+2^) and the associated generation of hydrogen peroxide in the presence of atmospheric oxygen or water (Tamayo et al., 2016)]. Interestingly, this is the same biological mode of action when using high-proof alcohol (arguably one of the most used method of sterilizing surfaces) (Palmer, 2010).

The need to address the issue of antimicrobial resistance due to broad use and abuse of antibiotics is very critical because of increased occurrence of multi-drug resistant bacterial strains which contribute to the reemergence of infectious diseases and persistent spread of hospital acquired infections. Antimicrobial resistance develops either through new mutation and selection in previously susceptible species, or by horizontal gene transfer during conjugation, transduction, and transformation in which new genetic materials are acquired from other resistant organisms (McManus, 1997). The new sets of antibiotics developed in the last decade have the same mechanism of antibacterial action as older antibiotics and have not been able to decrease the incidence of antibiotic resistance (Quinteros et al., 2016). This has necessitated the adoption of alternative strategies to treat bacterial infections rather than depending completely on conventional antibiotics which also have the limitation of low bioavailability and poor penetration at sites of infection (Canaparo et al., 2019). Metal nanoparticles can also be used as delivery agents when functionalized with a conventional antibiotic. In addition to combating antimicrobial resistance, this can also improve antimicrobial treatment efficiency, increase drug concentration at infection site, decrease bioaccumulation and decrease the risk of toxicity to nontarget tissues (Miller et al., 2014).

The application of nanotechnology in the fight against antimicrobial resistance could proffer solutions to some of the critical factors responsible for the persistence of antimicrobial resistance (Shaikh et al., 2015). Metals such as silver and copper have antibacterial properties in the bulk form and can be used as alternative forms of antibacterial therapy in nano-formulations which have unique physicochemical properties (Morones et al., 2005; Ruparelia et al., 2008; Varshney et al., 2012). The large surface area to volume ratio of nanoparticles creates active sites for attachment with different functional groups in a biological environment (Wang et al., 2006; Ezeuko et al., 2021). Nanoparticles serve as excellent candidates for drug delivery when paired with active pharmacological ingredients (Patra et al., 2018; Ezeuko et al., 2021). If nanoparticles are a viable alternative to conventional antibiotics, then progress must be made to ensure responsible development of engineered nanoparticles at environmentally friendly concentrations. For example, it is postulated that the ideal use of nanoparticles in agricultural, medical, and environmental applications is administration in lower doses because at higher doses, unintended consequences may become a significant factor (Roguska et al., 2015; Khan et al., 2016; Graham et al., 2017). In fact, there are discrepancies in literature when assessing the effectiveness of metal nanoparticle toxicity to bacteria (and other microorganisms). Most published reports focus on very high concentrations in an effort to prove efficacy (Roguska et al., 2015). However, we hypothesize that if nanoparticles are designed to specifically increase cytotoxicity in bacteria, while other environmental and health effects are reduced, then the doses needed to demonstrate metal-based nanoparticle antibacterial activity can be lowered.

This study reports a systematic investigation designed to deconvolute the relative bacterial inhibition of the physicochemical properties of four different engineered metal nanoparticles. We used minimum inhibitory concentration (MIC) coupled with minimum bactericidal concentration (MBC) and viable count assays to study the nanoparticle sphere (silver versus copper), surface coating (*a.k.a.* stabilizing agent; CTAB versus PVP), surface charge (positive versus neutral), and dose–response.

## Materials and methods

The sample naming convention used in this study is as follows: silver nanoparticles coated with CTAB are termed ‘CTAB-AgNPs’, silver nanoparticles coated with PVP are termed ‘PVP-AgNPs’, copper nanoparticles coated with CTAB are termed ‘CTAB-CuNPs’, and copper nanoparticles coated with PVP are termed ‘PVP-CuNPs’. In addition, the following controls were used: ‘0.4% CTAB’ representing the estimated concentration of CTAB in the CTAB-stabilized nanoparticles and ‘0.4% PVP’ representing the estimated concentration of PVP in the PVP-stabilized nanoparticles. These controls were used to parse the impact of the stabilizing agents independent of the nanoparticles. Lastly, the experimental design included a ‘no treatment’ control.

### Experimental design

**Figure 1:**
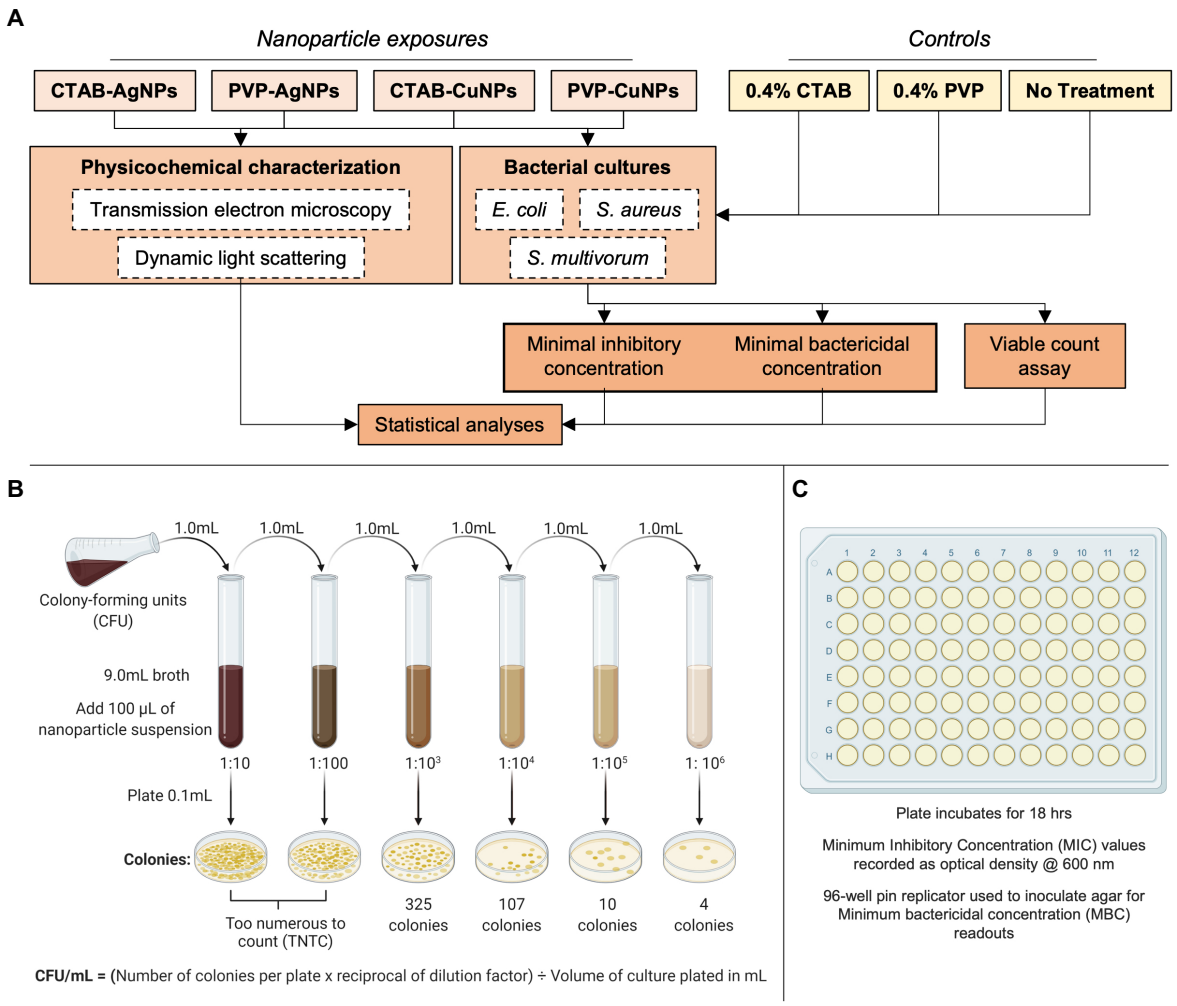
Experimental design used in this study. **(A)** The flowchart summarizes the experimental parameters used in study inclusive of nanoparticle-types (CTAB surface stabilized Ag, PVP surface stabilized Ag, CTAB surface stabilized Cu, and PVP surface stabilized Cu), controls materials (0.4% CTAB, 0.4% PVP, and no treatment), physicochemical characterization techniques (TEM and DLS), bacterial species (*E. coli*, *S. aureus*, and *S. multivorum*), and endpoint analyzes. The assays used to draw conclusions are depicted in **(B)** as viable plate count assay and **(C)** as minimum inhibitory concentration (MIC) and minimum bactericidal concentration. Figures 1B,C adapted from BioRender (https://biorender.com/).

The design of experiments used in this study are included in Figure 1. The project employed an interdisciplinary approach to collect data and interpret results. Each data set was verified using a secondary method to confirm observations, for instance, quantitative assessments of transmission electron microscopy images were compared to quantitative data obtained from dynamic light scattering. Data collected from minimum inhibitory concentration measurements were compared to data collected from the viable count assay. Each experiment was performed in triplicate with at least three replicates of each sample at the time of study.

### Reagents

Trisodium citrate (Na_3_C_6_H_5_O_7_, CAS# 6132-04-2, anhydrous) was purchased from Alfa Aesar (Haverhill, Massachusetts, United States) and silver nitrate (AgNO_3_, CAS# 7761-88-8, >99.9%) was purchased from Ricca Chemical Company (Arlington, Texas, United States). Polyvinylpyrrolidone (PVP, CAS# 9003-39-8, MW 40,000), sodium hydroxide (NaOH, CAS# 1310–73), copper (II) nitrate trihydrate (Cu (NO_3_)_2_·3H_2_O, CAS# 10031–43-3), and ethanol (EtOH, CAS# 64–17-5) were purchased from Sigma-Aldrich (St. Louis, Missouri, United States). Cetyltrimethylammonium bromide (CTAB, CAS# 57–09-0) was purchased from Bio-world (Dublin, Ohio, USA). L-ascorbic acid (C_6_H_8_O_6_, CAS# 50–81-7), copper (II) chloride dihydrate (CuCl_2_·2H_2_O, CAS#10125–13-0) and hydrazine hydrate (H_4_N_2_·H_2_O, CAS# 10217–52-4) were purchased from Acros Organics (Thermo Fisher Scientific, Waltham, Massachusetts, United States). Mueller Hinton agar was purchased from Oxoid Ltd. (Cheshire, England), while Mueller Hinton broth, nutrient agar, nutrient broth, and 0.5 McFarland standard were purchased from Remel (Thermo Fisher Scientific).

### Bacterial culture sources

Bacterial culture *Escherichia coli* (*E. coli*) 25,992 and *Staphylococcus aureus* (*S. aureus*) 6,538 were purchased from Microbiologics (St. Cloud, Minnesota, United States). *Sphigobacterium multivorum* (*S. multivorum*) 5,011 was isolated from Mexican fruit fly colonies.

### Nanoparticle synthesis

Silver nanoparticles stabilized with polyvinyl pyrrolidone (PVP) were synthesized in the dark by dropwise addition of 2 ml freshly prepared 5% silver nitrate (AgNO_3_) solution into 100 ml of 2% PVP solution under vigorous stirring at 100°C for 1 h (Wang et al., 2005; Samadi et al., 2010). Silver nanoparticles stabilized with cetyltrimethylammonium bromide (CTAB) were synthesized by dropwise addition of 50 ml freshly prepared 0.01 M AgNO_3_ solution into 50 ml of 0.01 M CTAB solution under vigorous magnetic stirring in one reaction vessel (Khan et al., 2009). Sodium hydroxide (NaOH; 50 ml of a 0.01 M solution) was added to 25 ml of 5.0 mM glucose solution in a different reaction vessel, followed by the addition of AgNO_3_-CTAB complex under vigorous magnetic stirring. The reaction was left for 5 h at 50°C until nanoparticle formation was complete.

Copper nanoparticles stabilized with PVP were synthesized by adding equal volumes of 0.8 M PVP and 0.4 M L-ascorbic acid (C_6_H_8_O_6_) mixture to 0.8 M PVP and 0.01 M copper (II) nitrate trihydrate Cu (NO_3_)_2_·3H_2_O mixture in a reaction vessel under vigorous magnetic stirring at 45°C (Wu et al., 2006). Nanoparticle formation occurs after a 3 h reaction time. Copper nanoparticles stabilized with CTAB were synthesized by adding equal volumes of 0.01 M CTAB and 0.08 M hydrazine hydrate (H_6_N_2_O) mixture to an equal volume of 0.01 M CTAB and 1.0 mM copper (II) chloride (CuCl_2_·2H_2_O), and the pH was adjusted to 10 under vigorous magnetic stirring for 3 h. For purification, all nanoparticle suspensions were centrifuged at 8000 rpm for 30 min to separate nanoparticles from unbound stabilizing agent molecules and the supernatant was stored at ambient temperatures. Pellets were re-suspended in known volume of ultrapure water prior to characterization.

### Nanoparticle characterization

Dynamic light scattering (ZEN 3690 Nanoseries Zetasizer; Malvern, Worcestershire, United Kingdom) was performed to measure the hydrodynamic diameter (nanoparticle size when suspended in aqueous phase) and zeta potential (indirect measure of nanoparticle surface charge when suspended in aqueous phase). Transmission electron microscopy (TEM) was performed for morphological characterization (JEOL, JEM-1010, TEM Tokyo, Japan). A drop of the prepared nanoparticle suspension was added unto the surface of carbon-coated copper grids (Electron Microscopy Sciences; Hartfield, Pennsylvania, United States) and dried in a hot air oven at 160°C. The dried TEM grids were then viewed on the TEM at an accelerating voltage of between 10 and 100 kV.

### Minimum inhibitory concentration

For all biological assays, a 2-fold serial dilution factor was used. The broth microdilution method was used to determine the minimum inhibitory concentration (MIC) of the surface-stabilized silver and copper nanoparticles on bacteria growth using flat bottomed 96-well plates covered with semipermeable clear adhesive and incubated for 16 h (Wiegand et al., 2008). Pure bacteria cultures were grown overnight and then adjusted to a concentration of 1×10^8^ CFU/mL using the 0.5 McFarland standard. Bacteria from these cultures were introduced to experimental wells containing 195 μl LB media and 5 μl of nanoparticles using a 96-well pin replicator. This assay had three main components: Luria broth, nanoparticle suspensions, and the bacteria species. Bacteria growth curves were collected using BioTek CYTATION5 plate reader. The protocol was adjusted to include an orbital plate shake step set for 7 s. Initial spectrum reading before incubation of plates and final spectrum reading after 16 h of incubation at 37°C were taken at OD_600_. The monitoring was performed based on a two-fold serial dilution of the nanoparticle systems at concentrations between 0.003 and 8 μM. Resultant MIC values were taken as the lowest concentration of the nanoparticle systems that inhibited visible growth of bacteria when observed with the unaided eye. Experiments were done in triplicate.

### Minimum bactericidal concentration

To determine the minimum bactericidal concentrations (MBC), Luria-broth agar plates were sub-cultured from the 96-well plates used in the MIC microdilution assay. The plates were incubated for 18 h and the MBC value was recorded as the lowest nanoparticle suspension concentration that reduced the viability of the initial bacterial inoculum by ≥99.9% as determined by visual inspection. The MBC represents the lowest concentration administered that resulted in the bacteria death.

### Viable plate count assay

All treatments including controls (i.e., no treatment, 0.4% CTAB, and 0.4% PVP) and the nanoparticle suspensions (CTAB-AgNPs, PVP-AgNPs, CTAB-CuNPs, and PVP-CuNPs) were tested using the viable plate count assay (Hoefel et al., 2003). Overnight cultures of *E. coli*, *S. aureus,* and *S. multivorum* were separately transferred into 10 ml of nutrient broth and incubated at 37°C (32°C for *S. multivorum*) for 18 h on a shaker at 100 rpm. Serial dilution of the resulting bacteria broth was done using 1: 1000,000 dilution factor. Each nanoparticle suspension (100 μl of 8 μM) was added to the serially diluted culture tubes and vortexed. Nanoparticle-treated bacteria suspension (100 μl) was pipetted onto a nutrient agar surface and spread evenly using culture spreader. The agar plates were incubated at 37°C (32°C for *S. multivorum*) for 18 h after which the colony forming units were counted to obtain the colony forming units per milliliter (CFU/mL) of nutrient media. In addition, the log_10_ reduction value relative to the no treatment group per bacteria species was also calculated for each experiment. Experiments were done in triplicate.

Statistical analysis of the nanoparticle effects on bacterial growth inhibition *via* the viable plate count assay was performed using analysis of variance (i.e., ANOVA) to fit a model that includes the following independent factors: stabilizing agents, nanoparticles, and bacteria species. Dunnett’s Multiple Comparison was used to test for deviation from controls for each stabilizing agent and each nanoparticle type. Tukey’s HSD test was used to identify differences between the three species. To estimate the effect of each independent factor (i.e., the proportion of variation explained by each factor), eta-squared, η^2^, was calculated using Equation 1:


(1) $$\eta^{2}=\frac{SS_{effect}}{SS_{total}}\;$$

which indicates large effects at 0.14, medium at 0.06, and small effect sizes at 0.01 (Cohen, 1988). All statistical tests were performed in JMP©, Version 13 (SAS Institute Inc., Cary, NC, 2016).

## Results

### Nanoparticle characterization

Table 1 shows data collected from dynamic light scattering (DLS). DLS analyzes provides three data sets for each nanoparticle characterized, namely hydrodynamic diameter (HDD), zeta potential, and dispersity index (DI). The HDD is the diameter of the nanoparticle plus stabilizing agent measured by speed. In other words, HDD is the hypothetical particle sphere that diffuses with the same speed as the particle being measured (Maguire et al., 2018). Table 1 reports that the HDD of PVP-AgNPs and PVP-CuNPs are both ~56 nm. We observed a difference in HDD between CTAB-AgNPs and CTAB-CuNPs, i.e., 76 versus 117 nm, respectively. Notably, all nanoparticles used in this study fit into the definition of engineered nanoparticles.

**Table 1:** Summary data collected from dynamic light scattering measurements.

Sample description	Sample ID	Hydrodynamic diameter (nm)	Zeta potential (mV)	Dispersity index (unitless)
Silver nanoparticles coated with polyvinylpyrrolidone	PVP-AgNPs	56.5 ± 2.09	−1.58 ± 0.13	0.496 ± 0.01
Silver nanoparticles coated with cetyltrimethylammonium bromide	CTAB-AgNPs	76.2 ± 1.12	+22.6 ± 0.35	0.314 ± 0.02
Copper nanoparticles coated with polyvinylpyrrolidone	PVP-CuNPs	56.1 ± 0.67	−0.39 ± 0.13	0.470 ± 0.02
Copper nanoparticles coated with cetyltrimethylammonium bromide	CTAB-CuNPs	117 ± 13.0	+24.3 ± 0.60	0.315 ± 0.05

The table includes measurements of hydrodynamic diameter, zeta potential, and dispersity index of the four nanoparticle types used in the study: silver and copper nanoparticles coated with polyvinylpyrrolidone (PVP), or cetyltrimethylammonium bromide (CTAB).

**Figure 2:**
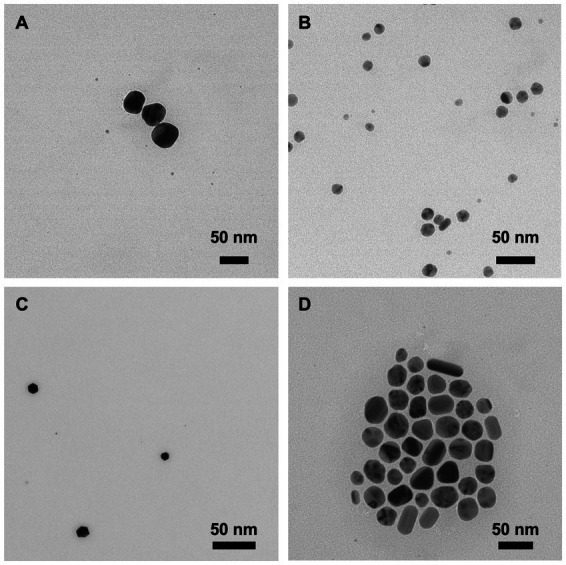
Transmission electron microscopy (TEM) images of surface stabilized copper and silver nanoparticles. **(A)** PVP-CuNPs **(B)** CTAB-CuNPs **(C)** PVP-AgNPs **(D)** CTAB-AgNPs.

The zeta potential is the electrical potential at the nanoparticle’s plane of shear and is often used to represent the particle’s surface charge (Bhattacharjee, 2016). PVP-AgNPs and PVP-CuNPs have a low electrical potential and can be considered effectively neutral; CTAB-AgNPs and CTAB-CuNPs produced a high electrical potential indicting a positive surface charge. The dispersity index (DI; *a.k.a.* dispersity index) specifies the or dispersity of the particle population. Each nanoparticle was synthesized with acceptable dispersity, meaning assurance of stability and HDD homogeneity. In addition, morphology of each nanoparticle is shown as transmission electron microscopy (TEM) images in Figure 2. CTAB-AgNPs exhibited a high degree of agglomeration, have a range of shapes from spherical to cuboidal, and have an average primary particle size of 84.6 ± 12.0 nm (Figure 2D). PVP-AgNPs are spherical, exhibit low particle agglomeration, and have an average primary particle size of 39.3 ± 6.7 nm (Figure 2C). CTAB-CuNPs are roughly spheroidal in shape with low particle agglomeration and have an average primary particle size of 71.7 ± 9.2 nm (Figure 2B). PVP-CuNPs are roughly spheroidal in shape, show moderate agglomeration in a chain-link formation, and have an average primary particle size of 38.8 ± 15.6 nm (Figure 2A).

### Minimum inhibitory concentration

**Figure 3:**
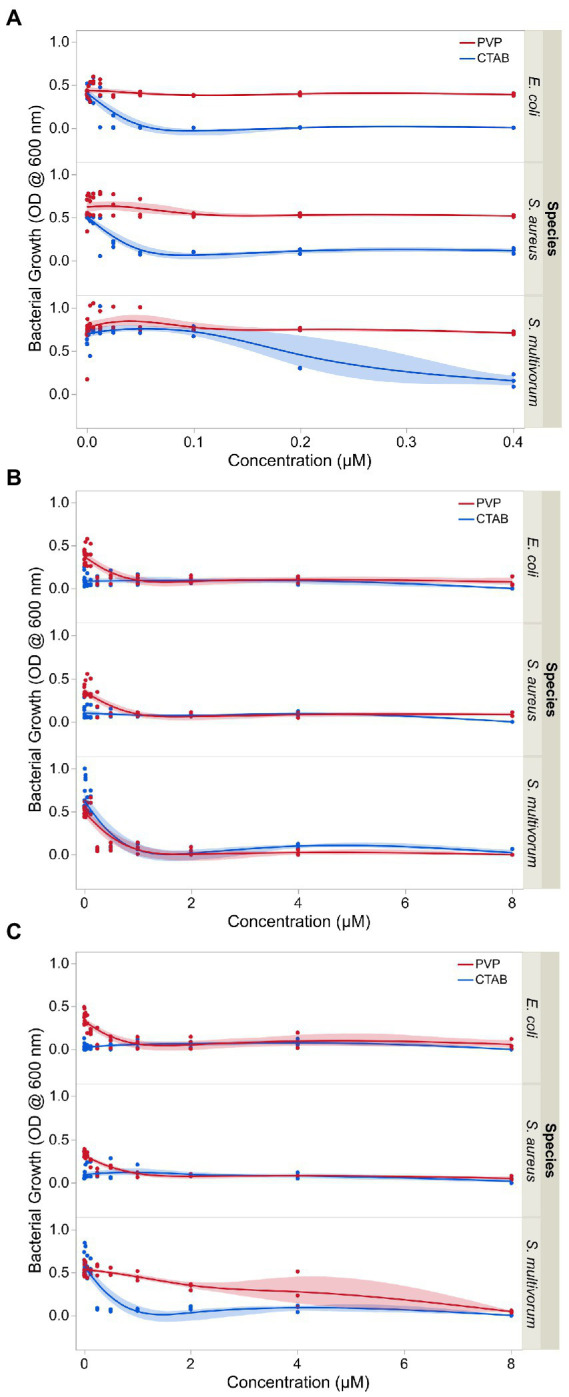
Growth inhibition of *E. coli*, *S. aureus*, and *S. multivorum* following exposure to varying concentrations of **(A)** surface stabilizing agents independent of nanoparticles, **(B)** surface stabilized silver nanoparticles, and **(C)** surface stabilized copper nanoparticles. The observations shown here were used to determine the minimum inhibitory concentration (MIC) of each treatment. Shaded areas indicate the confidence in the fit of the line.

The positively charged silver and copper nanoparticles showed the highest range of inhibition against the growth of the three bacteria species (Figure 3). Values for CTAB-AgNPs were 0.003, 0.003, and 0.25 μM for *E. coli*, *S. aureus* and *S. multivorum* while MIC values for CTAB-CuNPs were 0.003, 0.003, and 0.25 μM for *E. coli*, *S. aureus* and *S. multivorum*, respectively. MIC values of PVP-AgNPs 0.25, 0.125, and 0.25 μM for *E. coli*, *S. aureus* and *S. multivorum* while MIC values for PVP-CuNPs were 0.031, 0.125 and 2 μM for *E. coli*, *S. aureus* and *S. multivorum*, respectively.

### Minimum bactericidal concentration

**Figure 4:**
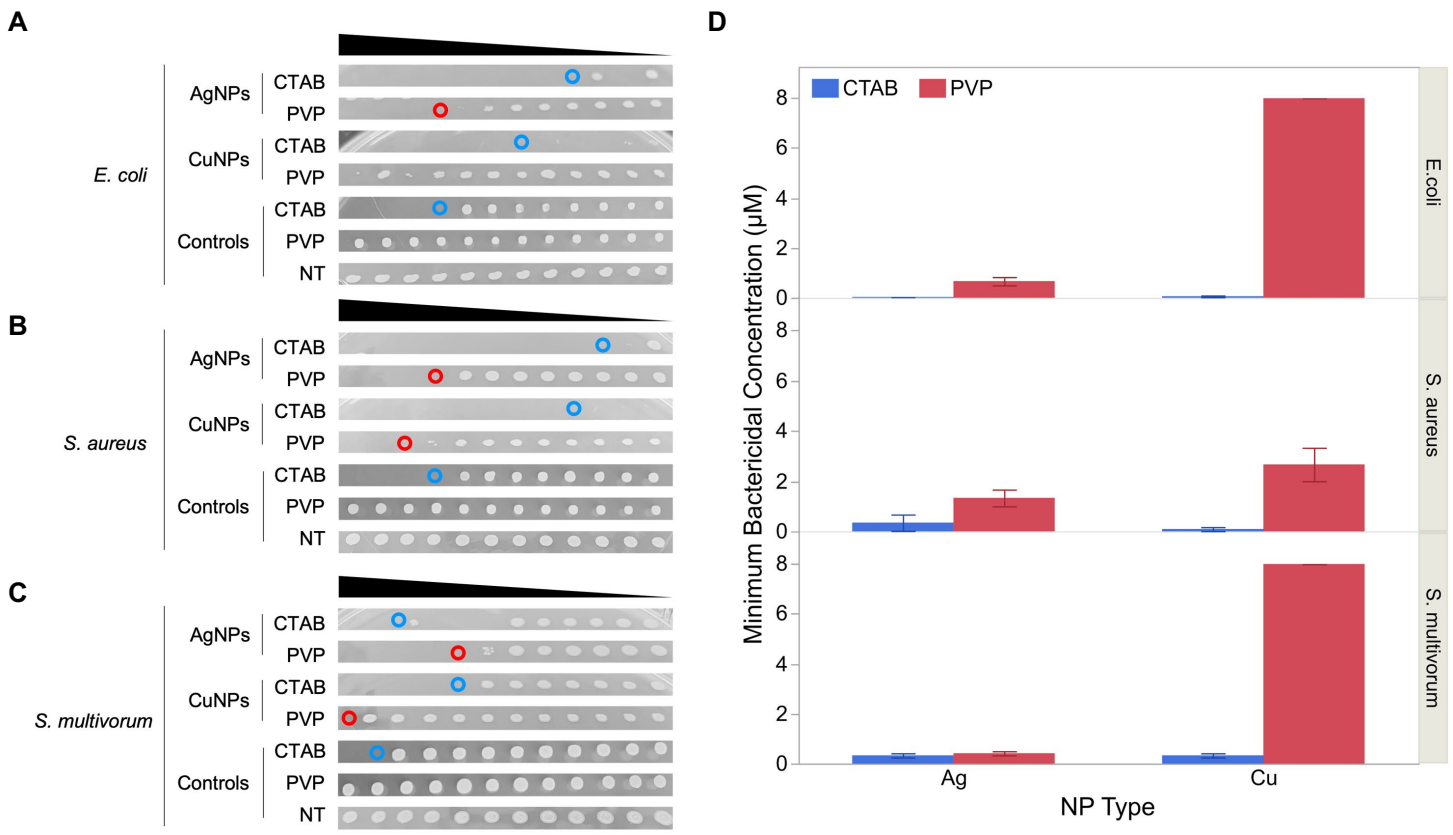
Minimum bactericidal concentration (MBC) of the four nanoparticles against the three bacteria-types used in this study. **(A)** Shows photographic evidence of effects against *E. coli*. **(B)** Shows the effects against *S. aureus*. **(C)** Shows the effects against *S. multivorum*. **(D)** Represents the average and standard error of the MBC value (*n* = 4). Within each panel, from top to bottom, each blot represents bacteria viability from the minimum inhibitory concentration (MIC) well-plates of each bacteria-type exposed to silver nanoparticles (AgNPs) stabilized with cetyltrimethylammonium bromide (CTAB), AgNPs stabilized with polyvinylpyrrolidone (PVP), copper nanoparticles (CuNPs) stabilized with CTAB, CuNPs stabilized with PVP, 0.4% CTAB control, 0.4% PVP control, and broth control. The black triangle above each panel represents decrease concentration from 2 μM serial diluted by 10.

Minimum bactericidal concentration (MBC) of each plate is shown in Figure 4. Values for CTAB-AgNPs were 0.031, 0.015, and 0.25 μM for *E. coli*, *S. aureus* and *S. multivorum* while MBC values for CTAB-CuNPs were 0.003, 0.015, and 0.25 μM for *E. coli*, *S. aureus* and *S. multivorum*, respectively. MBC values of PVP-AgNPs 0.5, 1.0, and 0.5 μM for *E. coli*, *S. aureus* and *S. multivorum* while MBC values for PVP-CuNPs were 8.0, 2.0, and 8.0 μM for *E. coli*, *S. aureus* and *S. multivorum*, respectively.

*S. aureus* exposed to PVP-CuNPs has a large standard error in the MBC results. This may be because the MBC blots did not clearly delineate the MBC value; there seems to be a tapering off effect (or gradient of bacteria cultures) and not a clear solid blot cut off.

Results show that CTAB is bactericidal; however, the interaction of CTAB with each nanoparticle produces a more potent bactericidal effect. Thus, CTAB-stabilized nanoparticles are more effective bactericidal agents. In contrast, PVP shows no sign of bactericidal action unless paired with the nanoparticles. But, PVP paired with nanoparticles, results in bactericidal effects. PVP-AgNPs are more effective than PVP-CuNPs across all concentrations tested. Bactericidal action from the MBC assay mirror bacteria growth inhibition patterns from the MIC assay.

### Viable plate count assay

Bacterial growth varied significantly between treatments [*F*_(6, 119)_ = 26.22, *p* < 0.0001, *R*^2^ = 0.88] and each main factor significantly contributed to variation in growth (*p* < 0.0001). Dunnett’s Multiple Comparison test showed that 0.4% CTAB, but not 0.4% PVP, significantly suppressed bacterial growth relative to the no treatment control (difference = 5.47 × 10^8^, *p* < 0.05). Both surface stabilized silver and copper nanoparticles significantly suppressed bacterial growth (silver difference = 1.09 × 10^9^, *p* < 0.05; copper difference = 6.71 × 10^8^, *p* < 0.05). Tukey’s HSD test showed that bacterial growth was significantly different between *E. coli* and *S. aureus* (difference = 4.55 × 10^8^, *p* = 0.0015) and *E. coli* and *S. multivorum* (difference = 5.51 × 10^8^, *p* < 0.0001), but not between *S. aureus* and *S. multivorum.*

**Figure 5:**
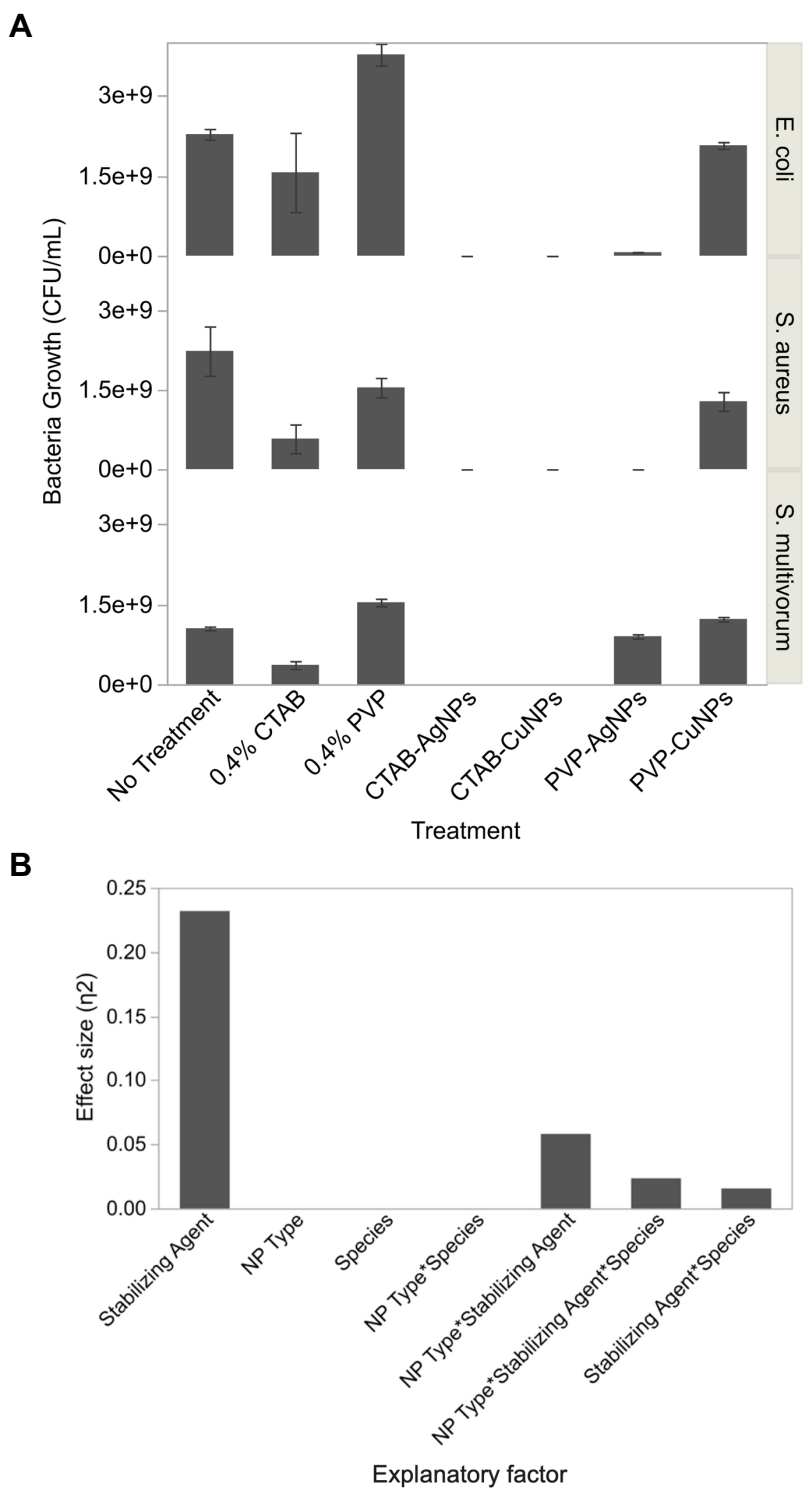
Colony forming units of bacteria growth obtained from viable plate count assay after exposure to surface stabilized silver and copper nanoparticles. **(A)** Bar graph showing the average and standard deviation of 3 replicates. **(B)** Effect size, or the proportion of variation in bacteria growth estimated by Eta squared, of each factor assessed in the viable count assay.

Interaction effects were more complicated (Figure 5A). CTAB-AgNPs and CTAB-CuNPs completely suppressed bacterial growth, while PVP-AgNPs produced a log reduction of 1.53 for *E. coli* (6.67 × 10^7^ CFU/ml), complete inhibition of *S. aureus*, and a log reduction of 0.07 for *S. multivorum* (9.0 × 10^8^ CFU/ml). Similarly, exposure to PVP-CuNPs resulted in a log reduction of 0.041 for *E. coli* (2.07 × 10^9^ CFU/ml), a log reduction of 0.241 for *S. aureus* (1.28 × 10^9^ CFU/ml), and log reduction of-0.07 for *S. multivorum* (1.22 × 10^9^ CFU/ml). Exposure to 0.4% CTAB treatment showed log reduction in bacterial growth of 0.163 for *E. coli* (1.56 × 10^9^ CFU/ml), log reduction of 0.588 for *S. aureus* (5.77 × 10^8^ CFU/ml), and log reduction of 0.461 for *S. multivorum* (3.63 × 10^8^ CFU/ml). Exposure to 0.4% PVP treatment showed log reduction of-0.218 for *E. coli* (3.77 × 10^9^ CFU/ml), log reduction of 0.160 for *S. aureus* (1.54 × 10^9^ CFU/ml), and log reduction of-0.165 for *S. multivorum* (1.54 × 10^9^ CFU/ml).

Eta-squared analysis is shown in Figure 5B. The stabilizing agent accounts for the largest proportion of variation in the formation of viable bacteria colonies. Nanoparticle-type (NP type) and bacterial species (species) play a smaller proportion of variation in the formation of viable bacteria colonies through their interactions with stabilizing agent.

## Discussion

Synthesis of metal nanoparticles *via* chemical reduction is the most common method due to low cost and tailorable properties (Pham et al., 2012). During synthesis, nanoparticle physicochemical properties are controlled by varying the experimental parameters such as concentration of reactants, temperature, and reaction time. Synthesis of metal nanoparticles *via* chemical reduction method using the polymer polyvinyl pyrrolidone (PVP which confers a neutral charge onto the surface of the nanoparticles) and the surfactant cetyltrimethylammonium bromide (CTAB which confers a positive charge onto the surface of the nanoparticles) have been documented (Trigari et al., 2011; Pham et al., 2012; Verma et al., 2015). Surfactants and polymers have been employed as surface coating and surface stabilizing agents in the synthesis of metal nanoparticles because they protect the core of the nanoparticle from oxidation, prevent nanoparticle agglomeration due to electrostatic and steric repulsion, and are also used to control the particle size and shape (Sarkar et al., 2008; Pham et al., 2012; Roh et al., 2013). Nanoparticle characterization validated the presence of metal nanoparticles after the chemical reduction synthesis method. Transmission electron micrographs showed the shape and size of the particles while dynamic light scattering reported hydrodynamic diameter (HDD), zeta potential (ZP), and dispersity index (DI) (Zhang et al., 2020).

Stabilizing agents can inhibit bacteria growth. Cetyltrimethylammonium bromide (CTAB) inhibits bacteria growth. CTAB belongs to the class of quaternary ammonium compounds, and they are extensively used as antiseptics and disinfectants in residential and occupational settings due to their inherent bactericidal activities (Denyer, 1995; Liao et al., 2020; Ongwae et al., 2020). These quaternary ammonium compounds target bacteria cells *via* electrostatic interactions between the positively charged headgroup of the molecule and the negatively charged cytoplasmic membrane of bacteria cell. The electrostatic interaction initiates adsorption onto the bacteria cell followed by side chain permeation into the intermembrane region which causes cytoplasmic leakage and ultimate cell lysis (Jennings et al., 2015). Several studies have demonstrated that the surfactant CTAB has biocidal properties (Tamuly et al., 2013). Several outcomes have been shown to occur when bacteria cells are exposed to CTAB and other surface active agents, such as membrane adsorption, membrane disorganization, or release of cytoplasmic constituents into surrounding matrix (Salton, 1951; Salton et al., 1951). Silver and copper nanoparticles with CTAB induced more bacteria growth inhibition than CTAB alone. CTAB-AgNPs and CTAB-CuNPs decreased bacteria growth demonstrating that when CTAB was paired with the nanoparticles (either silver or copper), growth of bacteria colonies was substantially decreased or even eliminated in the case of *S. multivorum*.

The PVP-AgNPs and PVP-CuNPs were less effective in decreasing bacteria growth compared to the CTAB stabilized metal nanoparticles. In general, PVP-AgNPs were more effective than PVP-CuNPs and there were more viable colonies of *S. multivorum* exposed to PVP-CuNPs than the no treatment control. In contrast to the observed results here, other studies have shown high antibacterial efficacy of PVP-AgNPs but the antibacterial efficacy was attributed solely to the metallic silver core (Batista et al., 2018). In this current study, it was observed that the PVP stabilizing agent seemed to promote the growth of *E. coli* and *S. multivorum*; showing more viable bacteria colonies than the no treatment control group against these bacteria. This observation could be explained with the assumption that PVP acts as a growth promoting factor for bacteria. It has been shown that the high water-binding capacity of PVP aids the metabolism of cells by maintaining moisture content in the cell media and that PVP binds to bacteria toxins released into media during the stationary growth phase, thereby extending the time before they reach the death phase (Deaker et al., 2004; Biradar and Santhosh, 2018).

There were three independent assays used to ascertain the bacterial growth inhibition data. Results of all three assays confirm the findings. Table 2 summarizes the agreement among the assay results and provides a clear indication that CTAB stabilized metal nanoparticles are quite effective antibacterial agents and that *S. multivorum* was the most susceptible to nanoparticle antibacterial activity.

**Table 2:** Summary of antibacterial effects data for all nanoparticles tested across all assays used.

Nanoparticle type	Minimum inhibitory concentration (MIC)	Minimum bactericidal concentration (MBC)	viable count assay
*E. coli*			
CTAB-AgNPs	++	++	++
PVP-AgNPs	+	++	++
CTAB-CuNPs	++	++	++
PVP-CuNPs	+		
*S. aureus*			
CTAB-AgNPs	++	++	++
PVP-AgNPs	+	++	++
CTAB-CuNPs	+	++	++
PVP-CuNPs	+	+	+
*S. multivorum*			
CTAB-AgNPs	+++	+++	+++
PVP-AgNPs	+++	+++	+
CTAB-CuNPs	+++	+++	+++
PVP-CuNPs	+++		+

The marks are plus signs indicating that antibacterial effects were demonstrated. If no marks are present in the well, then no effect was observed. The more marks (+) indicate better performance. Cells that contain the same number of marks within a grouping indicates equivalent performance.

This study demonstrates the use of engineered metal nanoparticles as possible antibacterial agents. The recorded MIC and MBC values and viable counts of bacteria after exposure to surface stabilized particles show that they can be effective at low doses. Silver and copper nanoparticles have reactive surfaces when suspended in aqueous media and have been shown to produce reactive oxygen species (ROS) (Abdal Dayem et al., 2017; Ameh et al., 2022). These particles have demonstrated antibacterial action against a range of antibiotic resistant bacteria species such as clinical strains of methicillin resistant *S. aureus* and *E. coli* (Quinteros et al., 2016; Liu et al., 2020). Bacterial toxicity of metal nanoparticles has been reported to be dependent on a combination of factors such as dose, physical and chemical properties, and amount of bacteria (Hajipour et al., 2012). Nanoparticles that do not aggregate or agglomerate have more available surface area; more available surface area translates into more ROS generation, greater chance of bacteria cell membrane interaction, and increased levels of released metal ions in surrounding matrix (Pramanik et al., 2012).

The need to explore non-traditional methods of antibacterial therapy is critical due to the persistence in antimicrobial resistance and the utility of antimicrobial nanoparticles could be a beneficial tool in addressing this clinical problem of critical importance. The safety and efficacy of these nanoparticles should continue to be investigated *via* cell based *in vitro* pharmacokinetic studies to broaden the knowledge perspectives on their use in biomedical applications. Unknown and unintended consequences of the increased use of nanoparticles in the industrial, consumer and healthcare sectors pose key challenges to human and environmental health. Developing frameworks for assessing risk associated with nanoparticle exposure are integral tools for maintaining human health and environmental safety. The critical quality attributes of nanoparticles during their life cycle such as fate and transport parameters, human and environmental health hazard profile, best practices for safe production as well as use and disposal, must be considered when applying control measures to reduce the associated risk from nanoparticle exposure (Warheit et al., 2008). Thus, it is of utmost importance to develop quantitative methods of risk assessment for determining environmental levels of metal nanoparticles and the resultant health effects of nanoparticle exposure in order to manage and mitigate such risks.

## Conclusion

The antibacterial efficacy of surface-stabilized metal nanoparticles was assessed to determine minimum inhibitory concentration and minimum bactericidal concentration. The antibacterial effects revealed in this study are dependent upon type of stabilizing agent and the interaction between stabilizing agent and the metal composition of the nanoparticle. The surface stabilized nanoparticles induced antibacterial action at relatively low doses. We have now assessed the potential for nanoparticles to be used as antibacterial agents in two different ways: antibacterial effects as a function of nanoparticle surface charge and antibacterial effects as a function of nanoparticle stabilizing agent. These features cannot be teased apart, but the knowledge unveiled herein can aid in formulating decisions. There is a need to further explore this phenomenon by systematically testing stabilizing agents that all confer a positive surface charge (like that of CTAB) or a neutral surface charge (like that of PVP) to differentiate effects between stabilizing agent and surface charge.

## Data availability statement

The raw data supporting the conclusions of this article will be made available by the authors, without undue reservation.

## Author contributions

TA: conceptualization, methodology, investigation, writing–original draft, formal analysis, and visualization. KZ and JD: methodology, investigation, and validation. WB: conceptualization, formal analysis, writing–review and editing, and visualization. CS: conceptualization, formal analysis, resources, writing–original draft, writing–review and editing, visualization, supervision, project administration, and funding acquisition. All authors have read and agreed to the published version of the manuscript.

## Funding

This research was supported by a cooperative agreement (#AP19PPQS&T00C023) between USDA Animal and Plant Health Inspection Service and Baylor University.

## Conflict of interest

The authors declare that the research was conducted in the absence of any commercial or financial relationships that could be construed as a potential conflict of interest.

## Publisher’s note

All claims expressed in this article are solely those of the authors and do not necessarily represent those of their affiliated organizations, or those of the publisher, the editors and the reviewers. Any product that may be evaluated in this article, or claim that may be made by its manufacturer, is not guaranteed or endorsed by the publisher.
